# Vancomycin leading to lupus flare in an elderly lady: a case report

**DOI:** 10.4076/1757-1626-2-6293

**Published:** 2009-07-23

**Authors:** Divey Manocha, Cristian Del Carpio Tenorio, Fredrick Rose

**Affiliations:** Internal Medicine, Suny Upstate Medical UniversityNY 13210USA

## Abstract

Elderly lady underwent right eye surgery for vitreous clot removal. 72 hours later, she complained of pain, redness and swelling in operated eye. Endophthalmitis was diagnosed. She was started on piperacillin/ tazobactam and vancomycin. Evisceration was required. Coagulase negative *Staphylococci* were isolated and vancomycin was continued postoperatively. She was discharged on home intravenous vancomycin therapy. Admitted one week later with painful oral ulcers, fever and diffuse erythmatous body rash. Vancomycin and other medications were stopped. Investigations revealed elevated double-stranded deoxyribonucleic acid and anti histone antibodies. Skin biopsy was suggestive of lupus rash. She was started on 60 mg of oral prednisone, improved dramatically and discharged to home.

## Case presentation

We hereby present an interesting case of an elderly Hispanic lady with known history of SLE with paroxysmal Atrial Fibrillation on chronic coumadin therapy. She was taking only maintenance dose oral prednisone prior to admission for SLE. She developed right vitreous hemorrhage on Coumadin and underwent clot removal surgery. 3 days later, she complained of severe pain, redness and swelling in right eye. CT scan of orbits was suggestive of endophthalmitis. She was started on IV piperacillin/ tazobactam and vancomycin. Subsequently, she underwent evisceration of right eye. Intraoperative cultures were positive for coagulase negative staphylococcus and vancomycin was continued in the post operative period and at the time of discharge for a total duration of 2 weeks. During this hospital stay she was continued on maintenance dose of 10 mg of prednisone for SLE.

One week after discharge, she complained of weakness, dizziness and fever of 101 F at home. No complaints of chest pain, dyspnoea, nausea or vomiting. No complaints of dysuria, cough/sputum or diarrhea. At the time of presentation to hospital, she was febrile, tachycardic and normotensive. She had painful oral ulcers and rapidly spreading diffuse erythematous rash all over body. Rest of systemic examination was within normal limits. Vancomycin and all other medications except prednisone were stopped. Basic metabolic profile, urine analysis and white cell count were normal. Blood and urine cultures were sent, right eye was reevaluated for any residual infection. Auto immune panel for assessing the lupus activity was sent. It showed elevated Anti ds DNA (229 u/ml)/ Anti Histone (119 u/ml)/Anti SSA (1055 u/ml)/ Anti SSB (338 u/ml) while Anti sm/Anti JO1/Anti RNP/complement remained normal. She also underwent biopsy of skin lesions which showed irregular linear and granular deposition of IgG, IgA and kappa light chain along the dermoepidermal junction, which supported the diagnosis of lupus flare up. Her prednisone was increased to 60 mg/ daily. Within 2 days she showed defervescence of the symptom complex and was discharged successfully to home. Her steroids were tapered down to maintenance dose and she continues to be in remission. Her latest ds DNA antibody levels were 94 IU/ml (normal <99 IU/ml) in January 2009.

## Discussion

Vancomycin Hydrochloride is a chromatographically purified tricyclic glycopeptide antibiotic derived from *Amycolatopsis orientalis* [1]. It is active against *Staphylococci*, including *Staphylococcus aureus* and *Staphylococcus epidermidis* (including heterogeneous methicillin-resistant strains) and *Streptococci*.

Common adverse effects include nephrotoxcity, ototoxicity, neutropenia, Most common cutaneous side effect is red man syndrome [2]. It is characterized by flushing and itching in upper torso with hypotension in severe cases and is caused by histamine release from the mast cells. Others include Steven Johnson syndrome, vasculitic rash and toxic epidermal necrolysis [3]. Lupus flare is one of the very rare side effects, with isolated similar cases reported in world literature [4].

Proposed mechanism is that the drug serves as a substrate for myeloperoxidase, which is activated in polymorphonuclear neutrophils. This interaction causes the formation of reactive metabolites that directly affect lymphocyte function. Another is that the genetic differences in an individual’s P450 system causes drugs to be metabolized differently, which results in the generation of toxic metabolites that may facilitate autoimmunity [5].

Classical features include- more common occurrence in whites and older age groups, infrequent involvement of renal and CNS, antihistone antibodies are common in >90%. Complement levels may be normal [6]. Clinically it can be associated with fevers, myalgias, arthralgias and a rash. And it usually resolves after several weeks after discontinuation of the offending medication [7].

Treatment includes stopping the culprit drug and short course of corticosteroids if the lupus symptoms are too debilitating as was the case with our patient. Follow up includes lupus antibody panel, complement levels and urine analysis. Long term prognosis is good.

Our patient did receive piperacillin/ tazobactum in the beginning of first admission for a short duration, but it was discontinued postoperatively, with isolation of coagulase negative *Staphylococcus*. She developed lupus symptoms 7 days postoperatively on vancomycin monotherapy. The flare responded promptly to discontinuation of the same. Thus the probability of piperacillin as the offending agent remains low.

## Conclusions

Vancomycin is a potent antibiotic used in various life threatening infections. It may lead to lupus flare up very rarely. Timely recognition of symptoms and discontinuation of the drug is key complete recovery. Systemic steroids may be needed if initial symptoms were severe.

**Figure 1.**
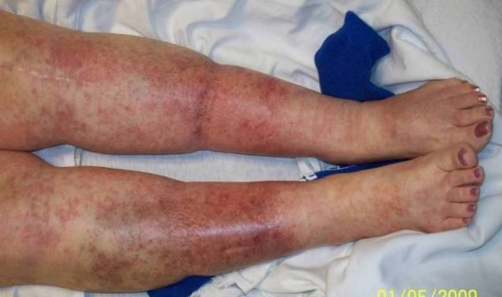
Anterior view of lower extremities showing the extension of the macular and petechial rash presented in our patient. Of note the right shin has signs of chronic venous stasis.

**Figure 2.**
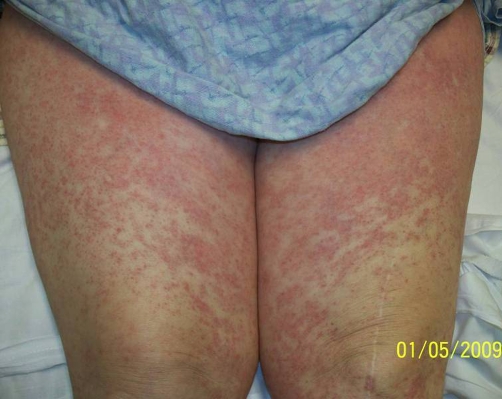
Anterior view of the thighs showing the macular rash in the patient, now slowly clearing.

**Figure 3.**
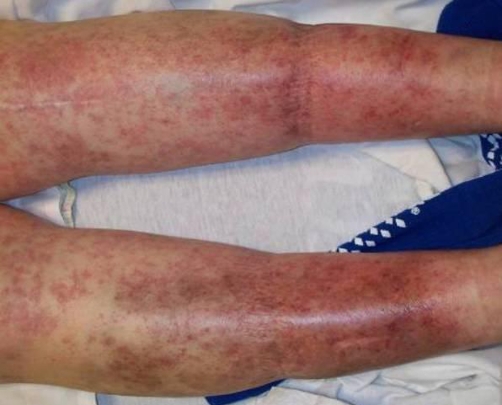
Posterior view of the lower extremities showing the macular and petechial rash present in the patient.

**Figure 4.**
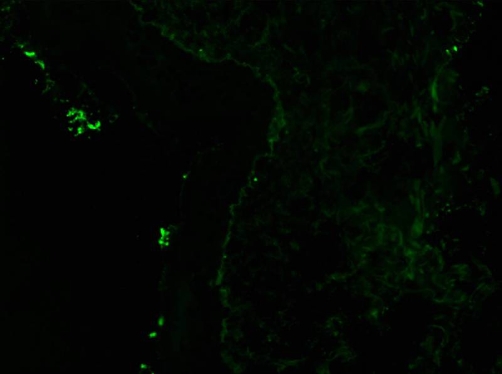
Immunofluorescence image showing IgA deposits in the along the dermo epidermal junction. For some technical reasons on photograph, IgG appears stronger than IgA, when the opposite was true on the real case.

**Figure 5.**
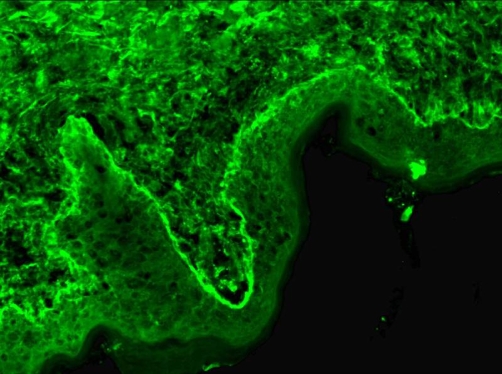
Immunofluorescence image showing IgG deposits in the along the dermo epidermal junction, which supported the diagnosis of SLE flare up.

## Abbreviations

F: Fahrenheit
IU: international units
IV: intravenous
Mg: milligram
SLE: systemic lupus erythematosus
U: units
